# On the Construction and Management of Hospitals for the Insane

**Published:** 1841-10-01

**Authors:** 


### ON THE CONSTRUCTION AND MANAGEMENT OF HOSPITALS FOR THE INSANE; WITH A PARTICULAR NOTICE OF THE INSTITUTION AT SIEGBURG. By Dr. *Maximilian Jacobi.* Translated by *John Kitching.* With Introductory Observations, &c. by *Samuel Tuke.* Octavo, pp. 299. London, Churchill, 1841.

THIS is an elaborate work on hospitals for the insane. It is quite a handbook on the subject, and should be in the possession of every one who has the management of such institutions. It would be out of our way to go into these matters at length, and we shall limit ourselves to noticing some points of general interest.

The Introduction by Samuel Tuke is written with much judgment, and with equally good feeling. A vein of sound benevolence, equally removed from cant and coldness, runs through it, and many of the suggestions contained in it deserve the attention of the public and Legislature.

*The state of pauper lunatics* appears to be any thing but wholesome. A large portion are confined in private houses, where they are of course received for profit; and in which it would be unreasonable to expect, that such an expenditure in proper buildings, in the enclosure of grounds for exercise, and in the provision of a sufficient number of competent attendants, should be made, as the circumstances of the disease render of the utmost importance. If all these things were provided in the manner most calculated to promote the recovery of the patients, it would not, in Mr. Tuke's opinion, be found that eight shillings a week, which is not the lowest price at which lunatic paupers are contracted for, would, to say nothing of medical attention, cover the charges of board, bedding, and clothing, in addition to rent and wages. It must be remembered that the cost of patients, as stated in the reports of our county asylums, does not include the items of rent and interest of capital.

Where a tenderer regard has been evinced, and a public provision made for the insane poor, it is far from what would be desirable. The Legislature has empowered the magistrates in Quarter Sessions, to erect suitable houses for the cure and care of the insane poor: they are authorised in these erections to make provision for other classes than paupers, and also to unite such establishments to any existing charitable institutions for the relief of the insane. Yet there are more than twenty English counties which have made no distinct provision for their insane poor.

*The surveillance of asylums* is any thing but perfect. There are no regularly appointed visitors. Mr. Tuke strongly recommends such, under the authority of Government, to inspect all the places of whatever description, private or public, chartered or unchartered, in which the insane are confined; to compare the degrees of human misery in these abodes— —to ascertain how it may be most effectually provided for and alleviated, —to collect information under uniform heads, from all these institutions, and to report, annually, to the public, the results of their observations and inquiries.

Mr. Tuke refers with satisfaction to Dr. Jacobi's advice, to restrict establishments for the insane to two hundred patients. If it be thought, he says, that the proper superintendence and medical care of this number is a sufficient charge for one individual, as the directing head, it does not appear that any argument in favour of large collections of lunatics, is to be drawn from the considerations of economy. A comparison of the cost of management in public schools of various sizes, has long led him to doubt, whether an establishment of any description, for three hundred persons or any larger number, will be managed *so* economically as one, containing from fifty to an hundred and fifty, would be likely to be. Every thing seems as if it must be done upon a magnificent scale in these large establishments, which of course swells the outlay; and from the returns made to Parliament of the average weekly cost of patients in the county asylums, it appears that Cheshire, a small establishment, is lower than Lancashire; that Norfolk is lower than Middlesex; and Suffolk than the West Riding of Yorkshire. Is it not also worthy of consideration, whether benefit would not be derived from diffusing the knowledge of the treatment of this disease more widely; which would be the consequence of establishing a larger number of hospitals for those who labour under it.

Mr. Tuke thinks that *the County Asylums should receive other besides pauper patients.* "Observation," he remarks, "in the York Asylum, in which patients of various classes in regard to property are received, convinces me that such an union of provision in one institution is in many respects advantageous. As there is no County Asylum for the North and East Riding of Yorkshire, many of the paupers in these districts are placed in this establishment; but I believe its service to the public has not been less, in having provided on moderate terms, and in a manner adapted to their condition, for the treatment of many patients of a class above paupers. The higher classes of patients who can afford to pay from twelve to twenty-one shillings per week, are of course a source of profit to the institution, and there have generally been so many in the house of this and a still higher class, as to cause the income from the patients to exceed the expenditure on their account, notwithstanding the much larger number which has been constantly admitted at less than eight shillings per week."

The highest class of society is not very likely ever to patronise County Asylums.

*Medical Attendance.*—The Asylums in England and Scotland, whether founded under the Act of Parliament or by voluntary charitable association, have generally a medical officer as the director of the establishment, and the medical treatment is entrusted to him, in conjunction with one or more non-resident physicians, who visit the patients twice or thrice a week. In a few establishments, the whole medical and moral management devolves upon the resident physician or surgeon. Mr. Tuke thinks that this does not work well. There ought to be the check, as well as the stimulus of a plurality of officers. If, for the purpose of obtaining more close medical attention, and a more uniform system of treatment, the plan of having outdoor officers is abandoned, there should be, at least, two resident officers who, though not equal in authority, should be united in the consideration of the plan of treatment. But Mr. Tuke rather leans to the employment of one resident medical officer, and a non-resident physician. The arrangement at the Siegburg institution is remarkable. For the care of two hundred patients, there are three resident medical officers, two ministers of religion, two stewards who have more or less intercourse with the patients, and also three superior attendants, who rank next to the officers of the establishment, and have the immediate direction of thirty-six attendants, (of whom we shall speak hereafter,) who have the personal care of the patients and their apartments. Thus we have introduced into the asylum a very large proportion of sane, and also, it is presumed from their station, of intelligent persons, who are very frequently coming into contact with the patients and their attendants, and whose intercourse and oversight are likely in various ways to operate beneficially upon both these parties. But to make such a plan beneficial, there must be a full confidence in the use and adaptation of the means employed, and a supply of efficient, judicious, and conscientious instruments for the carrying it into effect. For our own parts we think this not a little overdone. "Too many cooks spoil the broth" in madhouses as well as better places. The consequence of so many officers being huddled together would probably be either neglect or squabbling.

Mr. Tuke observes:

"The last thirty years have certainly witnessed an extraordinary improvement, in regard to the principles on which our houses for the insane, private as well as public, are professed to be conducted. The general recognition of sound principles indicates that to a considerable extent they are brought into operation. We have still, however, I apprehend, many steps to take before it can be said that we are availing ourselves, whether through the influence which mind has upon body, or which body has upon mind, of all the means which are afforded us for the cure or alleviation of insanity. In this course we may derive many useful hints from our continental neighbours; and we may reasonably expect that as the wants and capabilities of the insane become more correctly appreciated, and the qualities of mind required to supply them are better understood by the public at large, that the friends of patients will not be satisfied without obtaining for them those provisions which will most tend to their recovery; and that the persisting demand will lead to the supply of a greater number of persons who, in the various departments of our asylums, are qualified for the delicate office of administering to disordered minds. This, it must be acknowledged, is our great desideratum: it is the character of the persons engaged more than the change of system, or the increase of the number of officers, which will effectually raise the condition of our asylums; and I would observe that, if officers should be introduced into our establishments, charged more especially with the moral treatment of the patients, we ought never to let this duty devolve exclusively upon them; *all* should have their share in it, who are charged with any portion of the care of the patients; but in an especial manner ought the resident manager to feel this as a most important part of his duty. And in the selection of such an officer, the qualifications for moral management, amongst which I would specify,—a ready sympathy with man,—and a habit of conscientious control of the selfish feelings and the passions, ought ever to be sought as carefully as medical skill. If a moral manager and religious instructor be chosen, he should be one who knows experimentally the religion of the heart, who can condescend to the weak and the ignorant, and who, in the best sense of the phrase, can become all things to all men. I have observed, that the most successful managers of the insane have been those who were most humble and unselfish; and it is only persons of this class who will ever effectually supply their intellectual and religious wants.

A person of an opposite description, however talented, or however conversant with the philosophy of mind, or the doctrines of religion, can never exercise efficiently this divine art of healing." xx.

*Classification of Patients.*—Mr. Tuke of course recommends this. Dr. Jacobi proposes the following arrangement. 1. The raving and violent patients. 2. The noisy, whatever may be the kind of their insanity. 3. The dirty unbridled patients, or those sunk in a deep state of sexual excitement, with whom the depraved idiotic and stupid may be associated, if at all admitted into the establishment. 4. Those whose propensities and habits make them hurtful companions. 5. The melancholy and suicidally inclined. 6. The quiet and decently behaved. 7. Along with the preceding class must be a subdivision for the convalescents. This is the arrangement which he proposes for the patients in general with reference to their state of mind, independently of rank or payment, which of course leads to other divisions on which it is not essential to dwell. It must be borne in mind, that this classification of the patients is to be maintained by a system of prompt change, according to the fluctuations of their mental condition.

It was proposed, in the Wakefield Asylum, to divide the patients into three principal classes. "1. Those who, according to their states of mind, their capability of self-control, and the degree in which they were likely to annoy or be agreeable to each other, are disposed to incoherent laughing and singing, and generally all those who are capable of very little rational enjoyment. 2. Those who are capable of a considerable degree of rational enjoyment. 3. The convalescents and the well behaved patients, who are most capable of common enjoyments. The first class of course was intended to include the idiotic and demented as well as those who were most subject to violent action; and for these additional means of separation were planned by the provisions of a set of apartments, with a gallery, in which the most offensive patients could be at any time separated from the others of the class to which they belonged. This may be said to constitute four divisions; and if the number of patients associating together, were properly limited, and labour were efficiently introduced, I am inclined to think that a more refined division is not needful in a pauper asylum. The melancholy were to be divided between the second and third classes, according to their state of mind; it being thought undesirable to put all the dejected patients together: and I cannot see any substantial ground for Dr. Jacobi's change of opinion, as regards the bringing of this class of patients into one miserable congregation. In regard to the third, or best class of patients, in any other than an asylum for the labouring classes, the provision of a distinct apartment, where a few of the most rational, whether strictly convalescent or not, could associate together for reading, writing, conversation, &c. would be highly desirable; but it was considered that, in the Wakefield asylum, most of the patients, who were in the best state of mind, would be engaged in labour; and a work-room was also provided, which might be said to constitute another division: and, as it was presumed, that several of this class would also be employed in assisting in the house and garden, there did not appear occasion to make further provision for their classification."

Of the *Introduction of Labour* into lunatic asylums Mr. Tuke speaks in high terms.

*Coercive Measures.*—Mr. Tuke observes that the degree in which personal restraint is required towards the patients, depends very much upon the character of the attendants. Many fits of excitement, or acts of violence, which appear to justify coercion, would be prevented by a little kind consideration and judgment. There are many ways which can hardly be specified, by which an attendant may provoke a patient; nor are the arts by which an irritable excitable mind is soothed, more easy of description. There is no doubt, that the restriction of the *power* of attendants, and the not allowing them to impose personal restraint, without the consent of the Superintendent, has a tendency to lead them to cultivate the arts of prevention; and it may now, he believes, be said to be established, that, under fair management, the number of patients subjected to any kind of mechanical restraint, either by day or night, will rarely exceed *five out of a hundred,* and sometimes no one out of this number will be found to require it.

But Mr. Tuke would appear to be far from inclined to dispense with coercion altogether. His observations on this head merit considerable attention.

"Certainly," he says, "there are cases in which the patient himself solicits it—where the reason struggles with the impetuous delusion, and finds even in a degree of restraint, which upon a man not insane would be no prevention to mischief, a help to self-controul, a check upon the specific mode of action to which the malign influence impels him. Such cases are not very common, neither are they very rare. I have witnessed them, in connexion with a strong disposition to strike others, as well as with an occasional rushing impulse to a particular means of self-injury. There are also cases in which no struggle of the reason is perceived, marked by a determined disposition to violent and dangerous action, or a strong active suicidal tendency, in which I apprehend some restraint upon the free action of the body must be imposed, either by the *passive* resistance of mechanical applications, or by the active coercion of human force. It may be said, that these violent paroxysms are seldom of long continuance; but they are sometimes of sufficient duration to weary the patience, exhaust the mental and even animal resources, and excite the feelings of fear, resentment, or disgust in the mind of the attendant. If the patient is not to be restrained by a strap, are there no other vulgar appliances within the attendant's reach by which he may *overawe* the unhappy subject of his care? I fear and believe there are; and that in the struggles which cannot fail occasionally to take place, fear may be excited, sufferings may be inflicted, far more distressing than those occasioned by the right application of mechanical restraint, with this additional disadvantage, that they are less open to public notice. Nothing is more to be deprecated in the management of the insane, than protracted struggles between them and their care-takers. In our large institutions, the attendants must be left to a very great extent, to carry out the directions of the superintendent in their own way and spirit, and this, our knowledge of attendants as a class, hardly justifies us in expecting, will even *generally* be the best. This liability to abuse and perversion, even under enlightened management deserves consideration, but if the system has to be carried out by a reluctant or inefficient officer, or is left mainly to the ordinary attendants, there can hardly be a doubt of its inexpediency. The law of brute force, by which men so constantly tend to accomplish their ends, acts through an almost infinite variety of means, and neither chains nor straps are absolutely essential to its most cruel application." xxxiv.

There are some very good remarks on the *construction of hospitals* for the insane, which we must pass over. Mr. Tuke makes some observations on the *statistics of insanity*. He reasonably doubts if much dependence can yet be placed on calculations connected with it. Now as to the *prevalence of insanity*. Setting aside many aprocryphal calculations, we arrive at one which promises most accuracy.

In the year 1836, in pursuance of an address to the Crown, returns were made by the overseers of the various parishes in England and Wales, of the *pauper* lunatics and idiots within their respective districts; and from these returns it appeared that there were 13,667 of these afflicted persons chargeable to their parishes;—being a proportion of very nearly one in each thousand of the whole population of 13,897,187, as returned in 1831. Mr. Tuke apprehends that we may consider the lunatics and idiots, reported to be chargeable to their respective parishes, as being drawn from nearly, if not quite, one-half of our whole population. If this be a correct estimate, and we may consider the higher and middle classes of society as equally liable to mental disease with the lower, we arrive at the conclusion that insanity prevails in England and Wales in the proportion of at least one in every five hundred of its inhabitants.

The result of the inquiry in Norway, is a proportion of one insane person to every five hundred of the population. If, as Mr. T. apprehends is the case, the provision for the insane has been extremely deficient in that country, and if consequently the disease has not claimed that attention which more extensive provision for it would induce; there cannot be a doubt, that as the provision increases, so will the standard of sanity advance, and the number of cases which are classed under the head of insanity, be increased.

In the Rhenish provinces, under the Prussian Government, an attempt has been made to estimate the number of lunatics, and the result has been a proportion of 1 in every 1000 of the population; but Dr. Jacobi, whose establishment as Siegburg is appropriated to the use of these provinces, is of opinion that the returns are very defective, and that the proportion may be estimated at least at 1 in 600.

In France no general census has been attempted. Dr. Esquirol, in 1824, estimated the proportion of the insane to the population in France, at 1 in 1750: but he has subsequently enlarged his conjectural estimate to 1 in 1000.

In Italy, in 1833, Dr. Esquirol estimated the proportion at 1 in 3,785 of the population: but the statistics of Italy, in regard to insanity, as well as the provision for its treatment, are much more imperfect than those of France.

*Mortality of Lunatics.*—Mr. Tuke thinks that the *diet* of lunatics has a great deal to do with the comparative mortality of various institutions. "Our old sagacious superintendent at the Retreat, George Jepson, had not been long in his office before he came to the conclusion that the insane placed under his care required a liberal diet; and I cannot doubt, that the acting upon this opinion has been one cause of the very small mortality in this institution, as compared with that of most others. The York Asylum, many years ago, adopted a similar course, and I know it is the opinion of my friend, Dr. Wake, the physician to the institution, that the improvement of the diet has tended materially to the reduction of the proportion of deaths. The annual average mortality, during the 44 years of the Retreat experience, has been 4.70 per cent. upon the numbers resident. At the asylum during the last 25 years the proportion has been 7.35, during the previous 37 years of its existence the proportion was 11 per cent. In the large asylum at Hanwell, the average mortality has been 11.69, that of the West Riding of Yorkshire, 16.16, and that of Lancaster, 18.01. The mortality of *the paupers* in the private asylums at and near London, is stated at 20.68 per cent. upon the numbers resident." Mr. Tuke again and again insists on the necessity for an uniform system of reporting at Hospitals for the insane. The subject, he says, would not be unworthy of a special consultation amongst the professional men who are devoted to this department of the medical art; and, if once a uniform plan of arranging the facts and experience of our establishments were laid down, there is little doubt that it would be generally adopted by them; an approximation, at least, to a fair comparison of their respective results would then be obtained; and, if tabular statements were accompanied by a fair representation of any circumstances which distinguish the several institutions, we should hardly fail in time to arrive at most important and valuable deductions.

This closes the introductory remarks of Mr. Tuke. The work itself will be found to contain a mass of information as to the architectural arrangements, and the whole economy of madhouses. We fear it would be stepping out of our province, to follow Dr. Jacobi through his carefully composed details. They will amply repay the perusal of all who are interested in the matter.

